# Detecting two *Schistosoma* circulating antigens – CCA and CAA – in urine and serum to improve diagnosis of human schistosomiasis

**DOI:** 10.3389/fpara.2024.1460331

**Published:** 2024-10-04

**Authors:** Pytsje T. Hoekstra, Claudia J. de Dood, Theresia Abdoel, Stan Hilt, Angela van Diepen, Katja Polman, Peter Kremsner, Lisette van Lieshout, Andrea Kreidenweiss, Ayola Akim Adegnika, Daniela Fusco, Tahinamandranto Rasomoelina, Mala Rakoto Andrianarivelo, Raphaël Rakotozandrindrainy, Rivo Andry Rakotoarivelo, Elisa Sicuri, Govert J. van Dam, Paul L. A. M. Corstjens

**Affiliations:** ^1^ Leiden University Center for Infectious Diseases (LUCID), Leiden University Medical Center, Leiden, Netherlands; ^2^ Department of Cell and Chemical Biology, Leiden University Medical Center, Leiden, Netherlands; ^3^ Mondial Diagnostics, Amsterdam, Netherlands; ^4^ Department of Public Health, Institute of Tropical Medicine, Antwerp, Belgium; ^5^ Institute of Tropical Medicine, University Hospital Tübingen, Tübingen, Germany; ^6^ German Center for Infection Research (DZIF), partner site Tübingen, Tübingen, Germany; ^7^ Centre de Recherches Médicales de Lambaréné, Lambaréné, Gabon; ^8^ Department of Infectious Disease Epidemiology, Bernhard Nocht Institute for Tropical Medicine, Hamburg, Germany; ^9^ Centre d’Infectiologie Charles Mérieux, Antananarivo, Madagascar; ^10^ University of Antananarivo, Antananarivo, Madagascar; ^11^ University of Fianarantsoa, Fianarantsoa, Madagascar; ^12^ ISGlobal, Hospital Clínic, Universitat de Barcelona, Barcelona, Spain

**Keywords:** schistosomiasis, diagnostics, circulating cathodic antigen (CCA), circulating anodic antigen (CAA), duplex test

## Abstract

**Background:**

Schistosomiasis is caused by infection with parasitic *Schistosoma* worms and affects more than 250 million people globally. The detection of schistosome derived circulating cathodic and anodic antigens (CCA and CAA) has proven highly valuable for detecting active *Schistosoma* infections, causing both intestinal and urinary schistosomiasis.

**Aim:**

The combined detection of CCA and CAA was explored to improve accuracy in detecting *Schistosoma* infections.

**Methods:**

Parallel detection of CCA and CAA was performed on two banked sample sets with matching serum and urine samples from *Schistosoma mansoni* (*Sm*) and *S. haematobium* (*Sh*) infected individuals using the non-concentration based lateral flow (LF) test comprising the sensitive luminescent up-converting reporter particle (UCP) technology.

**Results:**

Parallel detection of CCA and CAA increased the positivity rate for detecting both *Sm* and *Sh* infections compared to the detection of either antigen separately, demonstrating the added value of detecting both antigens in a single sample to confirm diagnosis, independent from the *Schistosoma* species. Significantly higher CCA concentrations in urine were observed in *Sm* infected individuals compared to *Sh* infected individuals, while serum CCA-concentrations were similar between species. CAA concentrations were higher in serum compared to those in urine, irrespective of species. When exploring the relationship of CCA and CAA in urine, the CCA/CAA ratio in *Sm* infected individuals was significantly higher than in *Sh* infected individuals, while no differences were observed in serum.

**Discussion and conclusion:**

Parallel detection of CCA and CAA via the UCP-LF platform showed added diagnostic value through an increased positivity rate for the detection of *Sm* and *Sh* infections, compared to only detecting either of the antigens. The combined and quantitative detection of CCA and CAA is indicative for identifying the infecting species, but needs further exploration.

## Highlights

- CCA and CAA were detected in parallel in urine and serum samples from *S. mansoni* and *S. haematobium* infected individuals.
- Urine CCA concentrations were significantly higher in *S. mansoni* infected individuals than in *S. haematobium* infected individuals, while urine CAA concentrations were similar.
- Serum CAA concentrations were generally higher than urine CAA concentrations.
- Detecting both CCA and CAA improved diagnostic value compared to detecting infection by either antigen separately.

## 1 Introduction

Schistosomiasis is a neglected tropical disease affecting more than 250 million people of which the majority reside in sub-Saharan Africa (WHO, 2023). The disease is caused by parasitic blood flukes of the *Schistosoma* genus. Using accurate diagnostic tests to correctly identify and treat those who are infected is crucial in order to successfully reduce schistosomiasis burden and to move toward elimination of schistosomiasis. Viable schistosomes release a range of antigens into the hosts’ blood circulation and detecting these antigens allows for accurate diagnosis of active infections. The two *Schistosoma* genus specific gut-associated antigens called ‘circulating cathodic antigen’ (CCA) and ‘circulating anodic antigen’ (CAA) are well acknowledged for diagnosing active infections in humans (Bergquist, 2013). CCA and CAA – both gut-associated glycoconjugates – are regurgitated by living *Schistosoma* worms into the hosts’ blood circulation (van Dam et al., 1996), and are cleared via the kidneys into the urine (van Lieshout et al., 2000) with little day-to-day variation (Polman et al., 1998). Both antigens are therefore detectable in the infected individual’s serum as well as in urine. CCA and CAA have been shown to be cleared within a few days to weeks after treatment with praziquantel (PZQ) (Kildemoes et al., 2017; Sousa et al., 2019; Hoekstra et al., 2020; Hoekstra et al., 2022a), making antigen detection a highly effective tool for treatment monitoring. The initial development of monoclonal antibody (mAb) based sandwich ELISAs resulted in sensitive and highly specific detection of CCA and CAA (Deelder et al., 1989; de Jonge et al., 1990; Deelder et al., 1994; Deelder et al., 1996; Polman et al., 2000).

Since early 2000, the anti-CCA ELISA has been transformed into a mAb based lateral flow (LF) assay, in which CCA is detected in a single drop of urine with high sensitivity and specificity. Subsequently, a point-of-care test was developed which detects CCA in urine (POC-CCA) and is commercially available via Rapid Medical Diagnostics (Pretoria, South Africa) since 2008. The POC-CCA test is a non-invasive, user-friendly, field-applicable and visually scored LF urine test. Even though CCA is excreted by all *Schistosoma* species, the POC-CCA test has been demonstrated to be particularly useful in detecting intestinal *Schistosoma* species (Ochodo et al., 2015; Coulibaly et al., 2011; Tchuem Tchuente et al., 2012; Colley et al., 2013; Coulibaly et al., 2013; Adriko et al., 2014; Danso-Appiah et al., 2016; Kabore et al., 2017; Bärenbold et al., 2018; Colley et al., 2020a). Test performance lacks sensitivity in detecting urogenital infections (Ochodo et al., 2015; Stothard et al., 2006; Ashton et al., 2011; Danso-Appiah et al., 2016; Sanneh et al., 2017), due to significantly lower urine CCA concentrations in case of a *S. haematobium* infection. However, in some *S. haematobium* endemic areas, the POC-CCA test was reported to be a valuable tool for field diagnosis of urogenital schistosomiasis (Ayele et al., 2008; Midzi et al., 2009; El-Ghareeb et al., 2016). Additionally, co-infection with *S. haematobium* does not seem to influence the accuracy of the POC-CCA for accurately detecting *S. mansoni* infections (Coulibaly et al., 2013). A few studies have pointed toward reduced specificity in specific populations, e.g. in pregnant women (Greter et al., 2016; Marti et al., 2020; Casacuberta-Partal et al., 2021), small children (Midzi et al., 2009) and individuals with urinary tract infections or hematuria (RMD, 2018), likely due to cross-reactivity with host components (Van Dam et al., 1994; Casacuberta-Partal et al., 2021). Other limitation of the test include the interpretation of the so-called ‘trace results’ (Bärenbold et al., 2018; Peralta and Cavalcanti, 2018; Clark et al., 2021; Graeff-Teixeira et al., 2021; Clark et al., 2022; Kabbas-Piñango et al., 2023) as well as recently observed batch-to-batch variations in test performance (Viana et al., 2019; Colley et al., 2020b; Colley et al., 2023; Kabbas-Piñango et al., 2023). However, the POC-CCA has been repeatedly reported as very useful for *S. mansoni* diagnosis in field settings in endemic countries and the test is currently being recommended by the WHO as a more user-friendly and more sensitive alternative tool to stool microscopy for mapping prevalence of intestinal schistosomiasis prevalence and for surveillance purposes in *S. mansoni* endemic areas (Bärenbold et al., 2018; WHO, 2022; WHO, 2023).

Detection of CAA was significantly improved by the introduction of a LF test platform combined with a unique and highly sensitive luminescent reporter label – up-converting reporter particles (UCP) – that improved sensitivity more than 10-fold compared to previous ELISA assays (Corstjens et al., 2008; Corstjens et al., 2014). An extraction step with trichloroacetic acid (TCA) was added, which leaves carbohydrate structures such as CAA (but also CCA) in the clear supernatant fluid while precipitating proteins, thereby increasing the specificity of the test. Furthermore, by including a concentration step (i.e. increasing the sample volume to be tested), the sensitivity of the UCP-LF CAA test was further improved and in experimental studies it has been shown that the test is able to accurately quantify CAA concentrations down to the level of a single worm infection (Corstjens et al., 2020). The UCP-LF CAA test has demonstrated high specificity and sensitivity for the detection of all *Schistosoma* species including *Schistosoma* hybrids and veterinary species (Knopp et al., 2015; van Dam et al., 2015a; van Dam et al., 2015b; Vonghachack et al., 2017; Clements et al., 2018; Sousa et al., 2019; Corstjens et al., 2020; Hoekstra et al., 2021). It is applicable to various sample types, including urine, serum, plasma and dried blood spots (Stete et al., 2018; Downs et al., 2015; de Dood et al., 2018; Corstjens et al., 2020). An advanced and robust version of the UCP-LF CAA test has become available, omitting the need for a cold chain and thus facilitating storage and worldwide shipment and its implementation in basic equipped central laboratories in endemic regions (van Dam et al., 2013; Corstjens et al., 2015; Corstjens et al., 2020).

In this study, we aimed to combine the best of two worlds by evaluating the parallel detection of both CCA (being particularly sensitive for intestinal schistosomiasis) and CAA (highly sensitive and specific for all *Schistosoma* species), with the overall aim to explore the diagnostic potentials of a single UCP-LF based CCA/CAA duplex test. In order to shed further light into the presence of CCA and CAA in *S. mansoni* and *S. haematobium* mono-infected individuals, we applied the non-concentration, laboratory-based UCP-LF technique to qualitatively and quantitively assess both antigens in banked urine and serum samples. Furthermore, we investigated differences in species-specific excretion levels of CCA and CAA and if this could potentially provide information regarding the schistosome species.

## 2 Methods

This study utilized two sets of paired serum and urine samples available from two previous epidemiological studies, one performed in a heavily *S. mansoni* (*Sm*) infected community in Senegal (Stelma et al., 1993; Polman et al., 1995), and one performed in a high *S. haematobium* (*Sh*) endemic area in Cameroon (Kremsner et al., 1994). The original studies received ethical approval according to local standards and guidelines in place at the time of the study (Stelma et al., 1993; Kremsner et al., 1994; Polman et al., 1995). Extensive information was provided to the communities involved in the studies and oral informed consent was obtained from all participants (or from their parents in case of children) before sample collection. Urine and serum samples have been stored at -20°C and -80°C, respectively, since their arrival in the Netherlands, for detection of circulating antigen testing by ELISA as part of the original study and for future testing and validation of circulating antigen detection assays. Selection criteria were having a confirmed *Sm* or *Sh* infection (i.e. egg positive) as well as the availability of both a urine and serum sample. Egg positivity was based on microscopy, either by Kato-Katz (duplicate 25-mg slides from two stool samples collected within a time interval of 1-2 weeks) for the detection of *Sm* infections (Stelma et al., 1993; Polman et al., 1995) or by urine filtration (urine samples collected on 3 consecutive days) for the detection of *Sh* infections (Kremsner et al., 1994). A total of 105 and 97 matching urine and serum samples were available from *Sm* and *Sh* infected individuals, respectively.

CCA and CAA concentration was determined in each serum and urine samples using in parallel the non-concentration, dry format of the UCP-LF CCA test and UCP-LF CAA test, respectively, as previously described (Corstjens et al., 2014; de Dood et al., 2018; Corstjens et al., 2020). Briefly, 50µl of urine was mixed with 10µl of 12% of trichloroacetic acid (TCA), incubated and centrifuged, while for serum 50µl of each serum sample was mixed with an equal volume of 4% TCA, incubated and centrifuged. Subsequently, for either the urine test or the serum test 20µl of the clear supernatant was added to microtiter plate wells containing UCP reporter particles labeled with anti-CCA or anti-CAA antibodies hydrated in 100µL LF assay buffer and incubated on a shaker at 37°C. After 1h, LF strips were added to the wells and incubated overnight, followed by scanning the strips using a multistrip benchtop reader (UPCON; Labrox Oy, Turku, Finland). Samples with known CCA/CAA concentrations were included as a reference standard to quantify individual antigen concentrations and to validate the lower limit of detection (cut-off), which was based on previously published results: 2,460 pg/ml for urine CCA and 1,140 pg/ml for serum CCA, 20 pg/ml for urine CAA, 30 pg/ml for serum CAA (de Dood et al., 2018; Corstjens et al., 2020), with maximum quantification levels of 600,000 pg/ml for CCA and 20,000 pg/ml for CAA, see also **Table 1**. The cut-off for CCA is based on biological background presence of CCA-like host antigens (i.e. Lewis-X trisaccharide units) in urine and serum (Van Dam et al., 1994; Polman et al., 2000), while the CAA cut-off is based on technical aspects of the assay (Corstjens et al., 2014; Corstjens et al., 2015; Corstjens et al., 2020). Samples with a concentration above the indicated cut-off were considered positive. The Chi-square test was used to compare proportions and to determine statistically significant differences between antigens (CCA and CAA), between sample types (urine and serum) as well as between sample sets (*Sm* and *Sh*) and the Mann-Whitney U-test was used to compare quantitative CCA and CAA levels between groups, using SPSS version 29 and GraphPad Prism Version 10.2.3. CCA/CAA ratios in urine and serum were determined in samples which showed detectable (above the cut-off) concentrations of both CCA and CAA (see also **Supplementary Figure 1**).

**Table 1:** UCP-LF test formats used in this study with antigen target and respective thresholds.

Test format	Sample used	Antigen target	Lower threshold	Maximum quantification level
UCCA*hT*10	Urine	CCA	2,460 pg/ml	600,000 pg/ml
UCAA*hT*17	Urine	CAA	20 pg/ml	20,000 pg/ml
SCCA10	Serum	CCA	1,140 pg/ml	600,000 pg/ml
SCAA20	Serum	CAA	30 pg/ml	20,000 pg/ml

Based on previously published work (de Dood et al., 2018; Corstjens et al., 2020).

UCP-LF, up-converting reporter particle lateral flow; *hT*, high percentage of trichloroacetic acid (i.e. 12%, this is a crucial part of the sample preparation); UCCA, urine circulating cathodic antigen; UCAA, urine circulating anodic antigen; SCCA, serum circulating cathodic antigen; SCAA, serum circulating anodic antigen.

## 3 Results

Urine and serum samples from individuals with a microscopy-confirmed *Sm* or *Sh* infection were analyzed for the presence of CCA and CAA by the non-concentration, dry format of the laboratory-based UCP-LF test.

### 3.1 Percentage positive based on CCA and CAA

**Figure 1:**
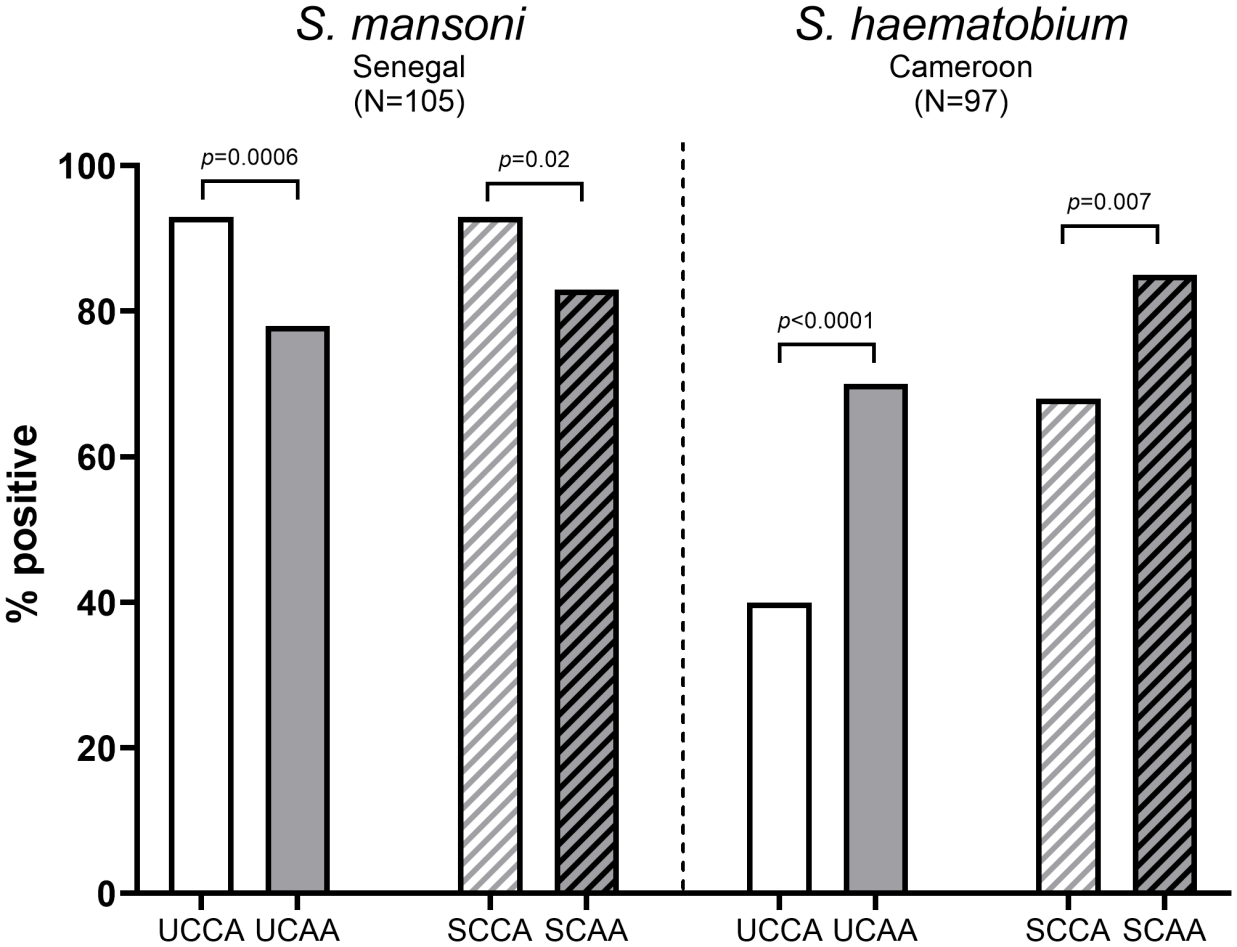
Proportion of CCA positive and CAA positive urine and serum samples from *S. mansoni* infected individuals (N=105) (left) and *S. haematobium* infected individuals (N=97) (right). Statistically significant differences between CCA and CAA are indicated in the figure by *p*-values. UCCA, urine circulating cathodic antigen; UCAA, urine circulating anodic antigen; SCCA, serum circulating cathodic antigen; SCAA, serum circulating anodic antigen.

CCA was significantly more often detected in *Sm* infected individuals compared to *Sh* infected individuals, in urine (93% *vs* 40%, respectively) as well as in serum (93% *vs* 68%, respectively). In contrast, the proportion of CAA positives was comparable between *Sm* and *Sh* infected individuals, in urine (78% *vs* 70%, respectively) as well as in serum (83% *vs* 85%), see also **Table 2** and **Figure 1**.

**Table 2:** CAA and CCA UCP-LF outcomes of urine and serum samples of *S. mansoni* infected (N=105) and *S. haematobium* infected (N=97) individuals.

	*S. mansoni* N=105	*S. haematobium* N=97
Egg microscopy*		
Mean egg count (SD)	720 (1,099)	490 (565)
Median egg count of the positives (IQR)	360 (90, 950)	256 (90, 732)
Urine		
CCA positive (%)	98 (93.3%)	39 (40.2%)
Mean CCA concentration in pg/ml (SD)	168,088 (215,450)	2,415 (3,070)
Median CCA concentration of the positives in pg/ml (IQR)	68,993 (21,761-272,307)	3,782 (3,027-6,031)
CAA positive (%)	82 (78.1%)	68 (70.1%)
Mean CAA concentration in pg/ml (SD)	740 (2,150)	638 (1,261)
Median CAA concentration of the positives in pg/ml (IQR)	350 (86-812)	607 (251-993)
CCA and/or CAA positive (%)	100 (95.2%)	71 (73.2%)
Serum		
CCA positive (%)	98 (93.3%)	66 (68.0%)
Mean CCA concentration in pg/ml (SD)	30,304 (94,301)	2,421 (2,430)
Median CCA concentration of the positives in pg/ml (IQR)	7,949 (3,395-20,459)	3,063 (2,002-4,530)
CAA positive (%)	87 (82.9%)	82 (84.5%)
Mean CAA concentration in pg/ml (SD)	8,048 (8,771)	8,730 (2,292)
Median CAA concentration of the positives in pg/ml (IQR)	5,991 (1,324-20,000)	7,599 (2,274-20,000)
CCA and/or CAA positive (%)	99 (94.3%)	90 (92.8%)
Urine & Serum		
CCA and/or CAA positive (%)	103 (98.1%)	90 (92.8%)

*Kato-Katz for *S. mansoni* and urine filtration for *S. haematobium*.

CCA, circulating cathodic antigen; CAA, circulating anodic antigen; IQR, interquartile range; SD, standard deviation.

#### 3.1.1 *S. mansoni* infected individuals

As demonstrated in **Figure 1**, urine- and serum-based testing for *Sm* infections were significantly more accurate for CCA than for CAA (urine CCA 93% *vs* urine CAA 78%, *p*=0.0006 and serum CCA 93% *vs* serum CAA 83%, *p*=0.02). Overall, CCA and CAA were equally well detected in urine and serum (CCA: urine 93% *vs* serum 93%, *p*=1 and CAA: urine 76% *vs* serum 83%, *p*=0.23). Combining CCA and CAA outcomes increased positivity in urine (from 93% and 78% to 95%) as well as in serum (from 93% and 83% to 94%).

#### 3.1.2 *S. haematobium* infected individuals

Contrary to *Sm*, for *Sh* infections, urine- and serum-based testing were significantly more accurate for CAA than for CCA (urine CAA 70% *vs* urine CCA 40%, *p*<0.0001 and serum CAA 85% *vs* CCA: 68%, *p*=0.007), see also **Figure 1**. CCA positives in urine were significantly lower than in serum (urine: 40% *vs* serum: 68%, *p*=0.0001), similarly to CAA (urine: 70% *vs* serum: 85%, *p*=0.02). Combining CCA and CAA outcomes increased positivity in urine (from 40% and 70% to 73%) as well as in serum (from 68% and 85% to 93%).

### 3.2 Intensity of infection based on CCA and CAA

**Figure 2:**
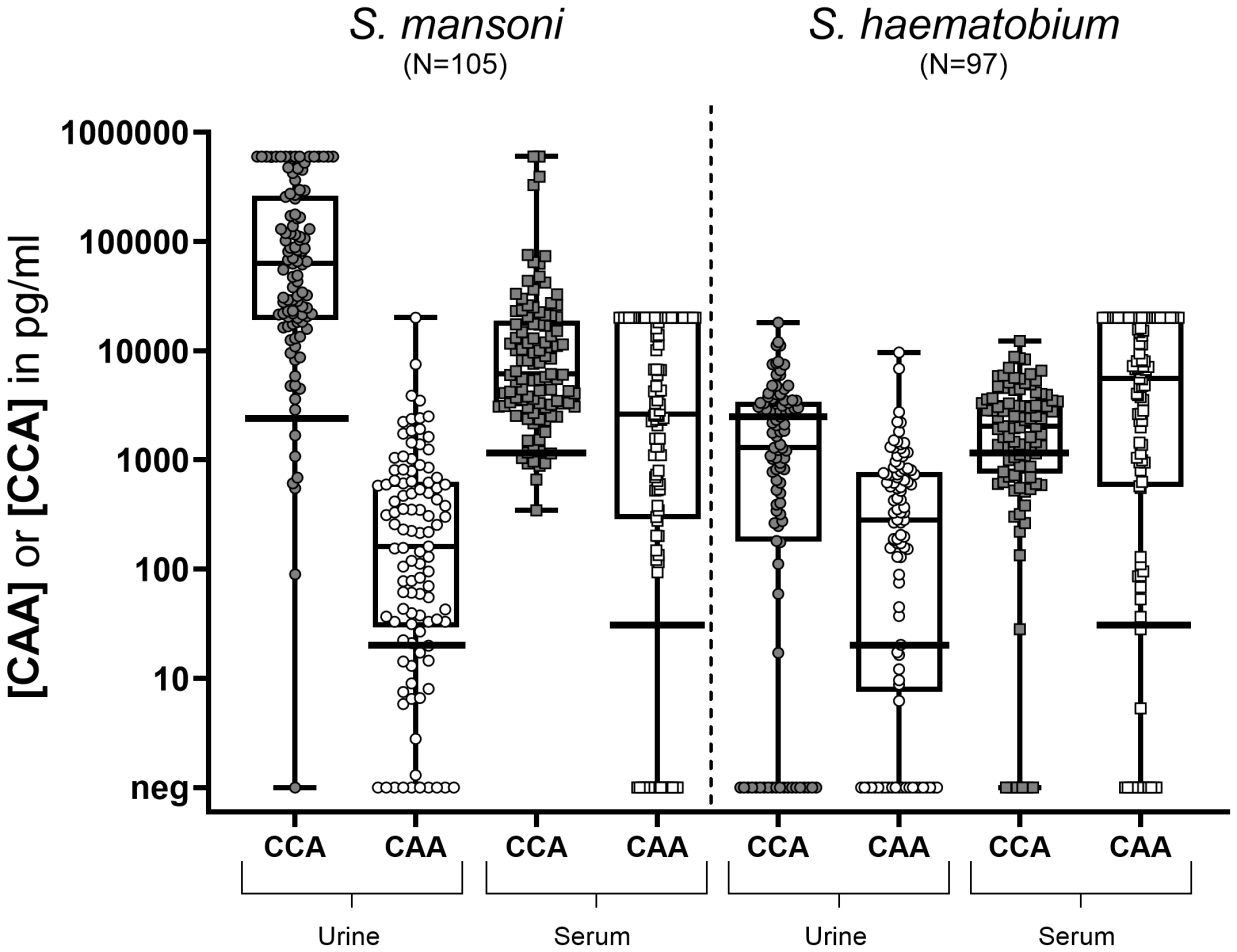
Box and whisker plot of individual CCA and CAA concentrations in urine (circles) and serum (squares) samples from *S. mansoni* infected individuals (N=105) (left) and *S. haematobium* infected individuals (N=97) (right). The box contains the 25^th^ to 75^th^ percentiles of the antigen concentrations, while the central lines denotes the median antigen concentration. The whiskers mark the minimum and maximum antigen concentration. The respective cut-offs (horizontal black lines) indicate the value above which a concentration is considered truly positive (i.e. 2,460 pg/ml for urine CCA; 20 pg/ml for urine CAA; 1,140 pg/ml for serum CCA and 30 pg/ml for serum CAA). UCCA, urine circulating cathodic antigen; UCAA, urine circulating anodic antigen; SCCA, serum circulating cathodic antigen; SCAA, serum circulating anodic antigen.

Being a measure of intensity of infection, CCA and CAA concentrations were quantitatively analyzed and compared between *Sm* and *Sh* infected individuals, both for serum and urine samples (**Figure 2**). In *Sm* infected individuals, CCA concentrations were significantly higher compared to CAA concentrations, in particular in urine but also in serum. In *Sh* infected individuals, CCA concentrations in urine and serum were lower than in *Sm* infected individuals, while urine and serum CAA concentrations were similar between species. In all individuals, CAA concentrations in serum were significantly higher compared to urine.

### 3.3 Identification of schistosome species based on CCA and CAA ratio

**Figure 3:**
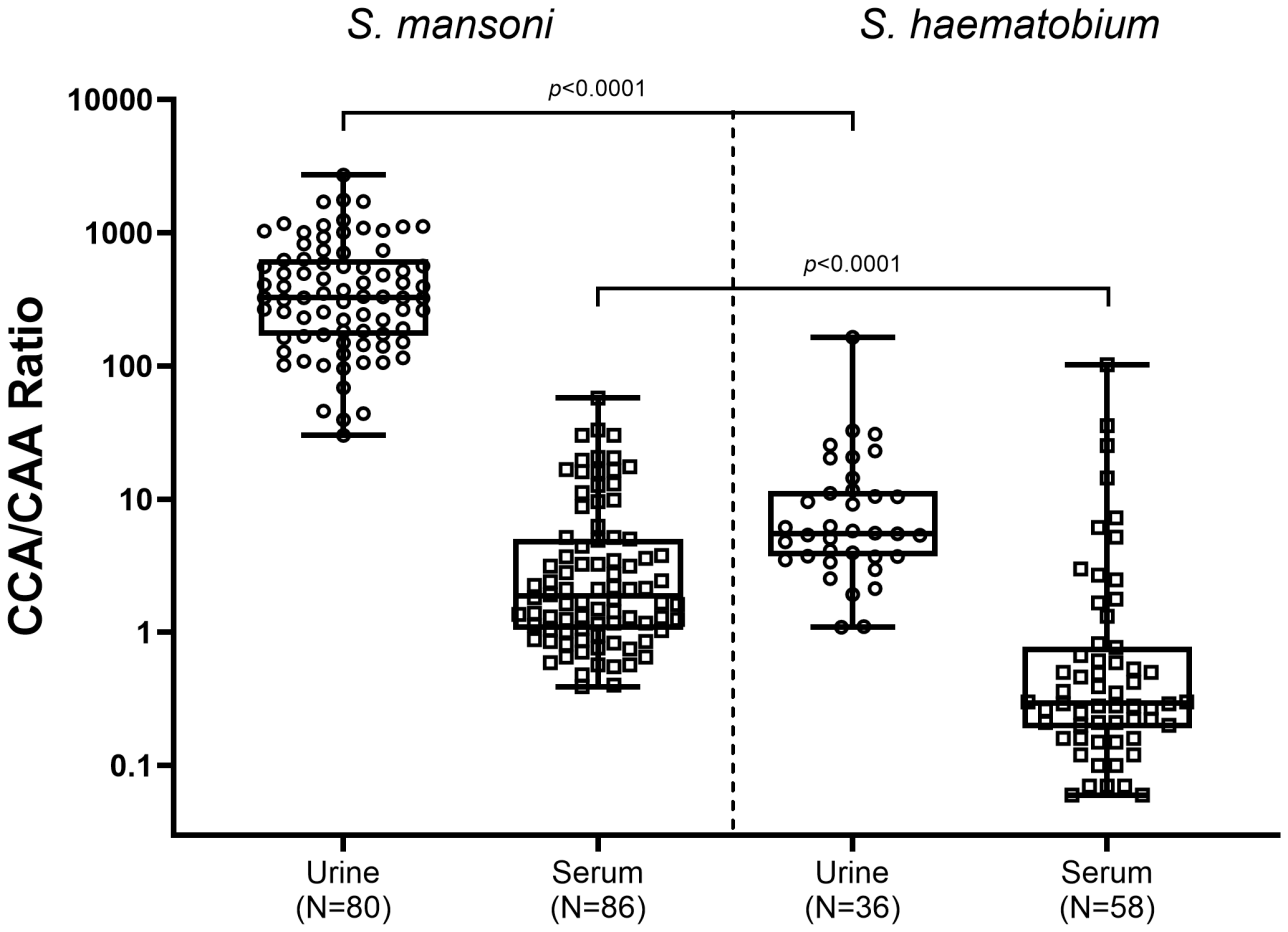
Box and whisker plot of individual CCA/CAA ratios in urine (circles) and serum (squares) samples from *S. mansoni* infected individuals (left) and *S. haematobium* infected individuals (right). The box contains the 25^th^ to 75^th^ percentiles of the CCA/CAA ratios, while the central lines denotes the median CCA/CAA ratio. The whiskers mark the minimum and maximum CCA/CAA ratio. CCA/CAA ratios were determined in urine and serum samples which showed detectable (above the cut-off) concentrations of both CCA and CAA (*S. mansoni*: N=80 urine and N=86 serum samples; *S.* haematobium: N=36 urine and N=58 serum samples. CCA, circulating cathodic antigen; CAA, urine circulating anodic antigen.

As significant differences in CCA and CAA concentrations between schistosome species were observed, the relation between CCA levels and CAA levels was analyzed (CAA/CCA ratios), in urine as well as in serum of *Sm* and *Sh* infected individuals (**Figure 3**). The highest CCA/CAA ratios were observed in urine of *Sm* infected individuals (median ratio of 327), being significantly higher than in urine of *Sh* infected individuals (median ratio 5.5, *p*<0.0001). Overall, CCA/CAA ratios in serum were lower compared to CCA/CAA ratios in urine. CCA/CAA ratios in serum of *Sm* infected individuals (median ratio 1.9) were also significantly higher than in serum of *Sh* infected individuals (median ratio 0.3, *p*<0.0001).

## 4 Discussion

This study is the first to thoroughly investigate the presence of two well-studied *Schistosoma* specific antigens – CCA and CAA – by utilizing the UCP-LF technique in matching urine and serum samples collected from *Sm* and *Sh* (microscopy-confirmed) infected individuals. The parallel detection of both CCA and CAA improved the positivity rate for detecting both *Sm* and *Sh* infections compared to detection of either antigen separately, confirming previous findings based on ELISA assays in *Sm* populations (Van Lieshout et al., 1992; Fillie et al., 1994), and thereby demonstrating the added value of detecting both antigens in a single sample for accurate diagnosis independent from the *Schistosoma* species. Furthermore, our results showed that quantitatively detecting both antigens can potentially provide information regarding the *Schistosoma* species, in particular identifying *Sm* infections in urine, provided that detectable concentrations (above the cut-off) are measured for both CCA and CAA.

Differences in CCA concentrations between the two *Schistosoma* species were observed: in *Sm* infected individuals CCA levels were significantly higher than in *Sh* infected individuals – with the greatest differences being observed in urine samples. Even though CCA is excreted by all *Schistosoma* species, the observation that the highest CCA concentrations (and highest CCA/CAA ratios) were observed in urines of *Sm* infected individuals confirm the usefulness of the currently available and WHO-recommended point-of-care (POC-CCA) test for diagnosing intestinal schistosomiasis (Bärenbold et al., 2018; WHO, 2022; WHO, 2023). In case of urogenital schistosomiasis, the median urine CCA concentration in this particular sample set was ca. 20 times lower, which explains that in particular for the low intensity infections, urine CCA concentrations most likely fall below the detection threshold of the POC-CCA test, resulting in lower diagnostic performance in these settings (Stothard et al., 2006; Ayele et al., 2008; Midzi et al., 2009; Stothard et al., 2009). This was confirmed in our study where, based on the more sensitive UCP-LF technique, detectable CCA concentrations were observed in urines from *Sh* infected individuals, although these concentrations were generally low and around the cut-off (**Figure 2**).

Overall, CAA concentrations were higher in serum compared to urine, irrespective of the *Schistosoma* species, which is similar to previous findings (Van Lieshout et al., 1995; van Dam et al., 1996; Polman et al., 1998; Polman, 2000; van Dam et al., 2015b; Sousa et al., 2019; Langenberg et al., 2020; Hoekstra et al., 2021; Casacuberta-Partal et al., 2022; Hoekstra et al., 2022b). The detection of CAA in serum is therefore highly suitable for diagnosing infection with all *Schistosoma* species. Currently, efforts are ongoing to develop a visually scored finger prick blood-based CAA rapid diagnostic test (CAA-RDT) in collaboration with FIND Dx (FIND, 2021). Combining the urine POC-CCA test with the upcoming finger prick blood CAA-RDT is expected to be a promising, field-friendly, diagnostic alternative for egg microscopy and would improve accuracy for diagnosing both *Sm* and *Sh* infections.

Based on species-specific excretion levels of CCA and CAA, the parallel detection of both antigens in serum and urine was hypothesized to be indicative for the infective species. In the current study, the urine CCA/CAA ratio in *Sm* infected individuals was significantly higher than the CCA/CAA ratio in *Sh* infected individuals, while in serum a similar trend but not a significant difference was observed. These findings indicate that if both CCA and CAA are detected in a urine sample and if the CCA/CAA ratio is high (>200 based on the results of our study), an infection with *Sm* is most likely, while no definitive conclusions can be drawn from serum. Although interesting as a rule of thumb, to further corroborate this conclusion, larger studies in different endemic settings and infection intensities are needed. Furthermore, antigen concentrations can vary from person to person and several factors should be taken into account when determining the CCA/CAA ratio, including the intensity of infection (i.e. worm burden) and individual antigen clearance mechanisms.

Limitations of this study include the pre-selection of the sample set based on the presence of *Schistosoma* eggs (in urine or stool, depending on the species). Some egg positive cases could not be confirmed with either CCA or CAA in urine and/or serum (~5% of cases). Discrepancies were observed over a range of different egg counts which should be further investigated by re-testing with higher sample volumes, which allows for the detection of a lower antigen concentration. As antigen diagnostics are in general more sensitive compared to microscopy, the opposite (egg negative and antigen positive) would be expected to occur more often, as also observed in previous studies (Knopp et al., 2015; Sousa et al., 2019; Hoekstra et al., 2020; Tamarozzi et al., 2021; Hoekstra et al., 2022a; Hoekstra et al., 2022b). Preferably, a larger sample set including egg positives as well as egg negatives from various schistosomiasis endemic settings should be explored, including areas where multiple species are prevalent and/or where hybrid infections occur. Because the available volume of urine and serum samples was limited, we were only able to apply the non-concentration format of the UCP-LF test. This format is less sensitive compared to the UCP-LF concentration format, resulting in lower accuracy when quantifying lower antigen concentrations. In case larger volumes are available, the most sensitive format of the UCP-LF CAA test should be performed, leading to a more accurate antigen measurement, in particular in the lower range around and below the currently used cut-off (Corstjens et al., 2020). On the other hand, in some cases the maximum level of detection (plateau value) was reached; this was mainly the case for CCA in urines from *Sm* infected individuals and CAA in serum from both *Sm* and *Sh* infected individuals. Ideally, these samples should be diluted and re-tested in order to obtain a more accurate quantitative measurement of the antigen concentration.

## 5 Conclusion

Parallel detection of CCA and CAA using the UCP-LF platform showed added value compared to detecting only one of the antigens. In particular in *S. haematobium* endemic areas, where a limited performance of the POC-CCA urine test is observed, additional detection of CAA – for example via the upcoming CAA-RDT – will result in a better estimation of the true prevalence of schistosomiasis. More studies are needed to confirm whether the quantitative detection of CCA and CAA can be used to specify the infecting species, thereby also including mixed and/or hybrid *Schistosoma* infections. Next steps include further optimization of the combined detection of both CCA and CAA in a single test format via the UCP-LF technique as well as to engage with industrial partners for potential commercialization of such a duplex test into a POC format for use in low-resource settings.

## Data Availability

The original contributions presented in the study are included in the article/**Supplementary Material**. Further inquiries can be directed to the corresponding author.

## freeBILy consortium

The freeBILy consortium consist of eight partners institutes including four from Africa and four from Europe, represented by the following members: G.J. van Dam, P.L.A.M. Corstjens, C.J. de Dood, P.T. Hoekstra, A.S. Amoah, M.I. Keshinro, S.T. Hilt, (LUMC); A. Kreidenweiss, (EKUT); N.G. Schwarz, D. Fusco, P. Klein, A. Jaeger, E. Lorenz, (BNITM); A.A. Adegnika, Y.J. Honkpehedji, J.C. Dejon-Agobe, R. Beh Mba, M. Mbong Ngwese, M. Nzamba Maloum, A. Nguema Moure, B.T. Meulah, J. Gerstenberg, R. Laclong Lontchi, (CERMEL); R.A. Rakotoarivelo, A. Ralaizandry, M. Radomanana, (UF); R. Rakotozandrindrainy, N. Rakotozandrindrainy, M.J. Solonirina, J. Randriamanjara, (UA); M. Rakoto Andrianarivelo, T. Rasamoelina, R. Razafindrakoto, (CICM); E. Sicuri, C. Aerts, and F. Roeder (ISGlobal).

## Supplementary material

Supplementary Figure 1 Comparing CCA versus CAA concentrations in urine (circles) and serum (squares) samples from *S. mansoni* infected individuals [**(A, B)**, N=105] and *S. haematobium* infected individuals [**(C, D)**, N=97]. The red dotted lines indicate the cut-off above which a concentration is considered truly positive based on the test formats used in this study (i.e. 2,460 pg/ml for urine CCA; 20 pg/ml for urine CAA; 1,140 pg/ml for serum CCA and 30 pg/ml for serum CAA).
