# Supplementary figures and images for: Detecting two Schistosoma circulating antigens – CCA and CAA – in urine and serum to improve diagnosis of human schistosomiasis
Source: Front Parasitol. 2024 Oct 4;3:1460331. doi: 10.3389/fpara.2024.1460331 (PMC11732024; doi:10.3389/fpara.2024.1460331)

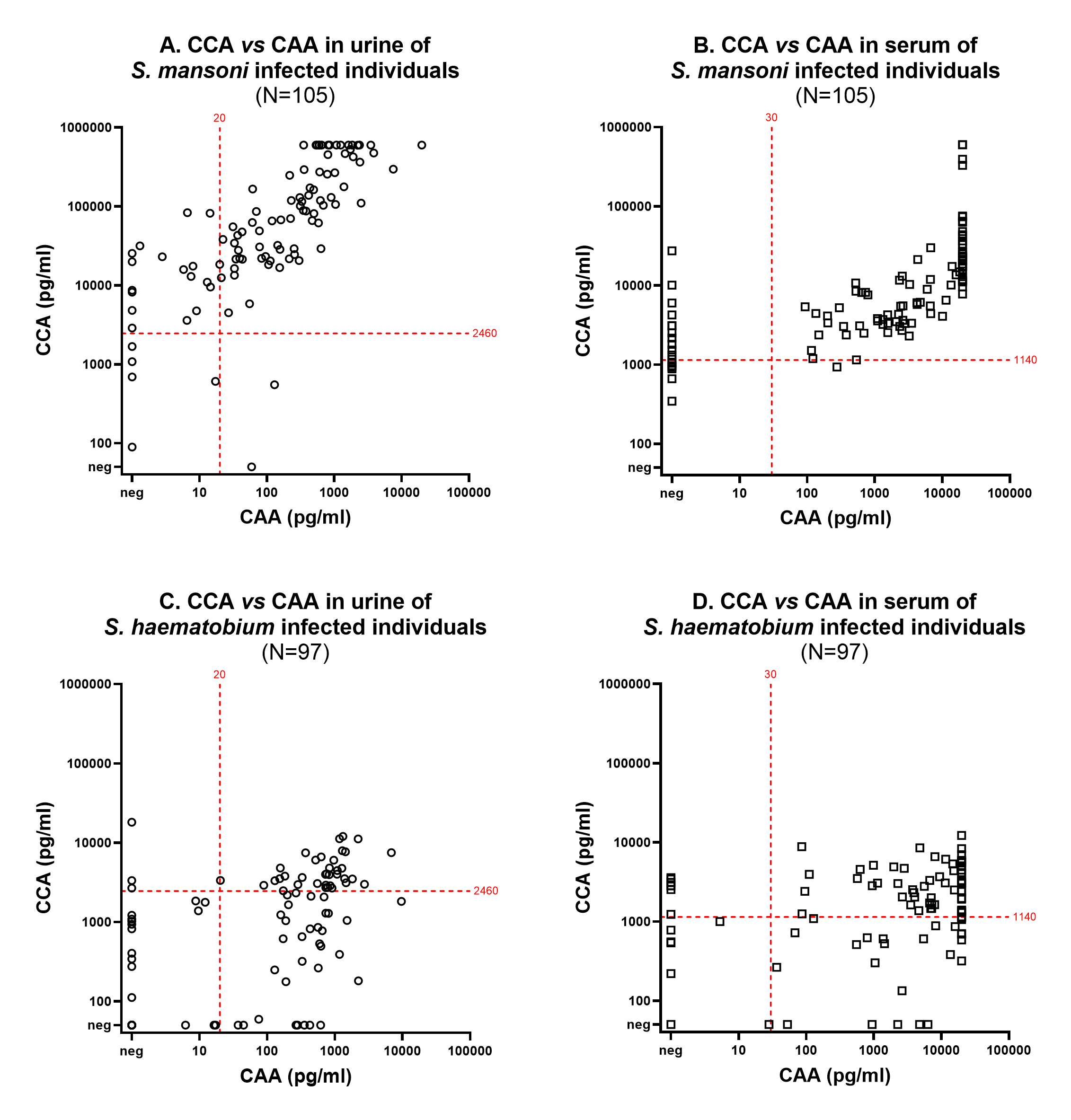

Supplement: Supplementary Figure 1 — Comparing CCA versus CAA concentrations in urine (circles) and serum (squares) samples from S. mansoni infected individuals [(A, B), N=105] and S. haematobium infected individuals [(C, D), N=97]. The red dotted lines indicate the cut-off above which a concentration is considered truly positive based on the test formats used in this study (i.e. 2,460 pg/ml for urine CCA; 20 pg/ml for urine CAA; 1,140 pg/ml for serum CCA and 30 pg/ml for serum CAA). [file Image1.tif]
